# Design of an Amphiphilic Poly(aspartamide)-Mediated Self-Assembled Nanoconstruct for Long-Term Tumor Targeting and Bioimaging

**DOI:** 10.3390/molecules24050885

**Published:** 2019-03-02

**Authors:** Kondareddy Cherukula, Saji Uthaman, In-Kyu Park

**Affiliations:** 1 Department of Biomedical Science and BK21 PLUS Centre for Creative Biomedical Scientists, Chonnam National University Medical School, Gwangju 61469, Korea; cherrikonda@gmail.com; 2 Department of Polymer Science and Engineering, Chungnam National University, 99 Daehak-ro, Yuseong-gu, Daejeon 34134, Korea; sajiuthaman@gmail.com

**Keywords:** polymeric nanoparticles, cyclic RGD, tumor targeting, long-term bioimaging

## Abstract

Biodegradable polymers have been developed for the targeted delivery of therapeutics to tumors. However, tumor targeting and imaging are usually limited by systemic clearance and non-specific adsorption. In this study, we used poly(amino acid) derivatives, such as poly(succinimide), to synthesize a nanomicelle-forming poly(hydroxyethylaspartamide) (PHEA, P) modified sequentially with octadecylamine, polyethylene glycol (PEG, P), and glycine (G) to design PHEA-PEG-glycine (PPG) nanoparticles (NPs). These PPG NPs were further tethered to cyclic Arg-Gly-Asp (cRGD) sequences for formulating tumor-targeting PPG-cRGD NPs, and then loaded with IR-780 dye (PPG-cRGD-IR-780) for visualizing tumor homing. cRGD cloaked in PPG NPs could bind specifically to both tumor endothelium and cancer cells overexpressing α_v_β_3_ integrins. PPG-cRGD NPs exhibited enhanced physiological stability, cellular viability, and targeted intracellular uptake in cancer cells. In addition, PPG-cRGD NPs offered enhanced systemic circulation, leading to preferential tumor targeting and prolonged fluorescence tumor imaging for nearly 30 days. Nevertheless, non-targeted formulations demonstrated premature systemic clearance with short-term tumor imaging. Histochemical analysis showed no damage to normal organs, reaffirming the biocompatibility of PHEA polymers. Overall, our results indicated that PPG-cRGD NPs, which were manipulated to obtain optimal particle size and surface charge, and were complemented with tumor targeting, could improve the targeted and theranostic potential of therapeutic delivery.

## 1. Introduction

Cancer remains one of the major causes of deaths worldwide. Intensive studies have been performed to improve traditional treatment strategies. The rapid emergence of nanoparticle (NP)-based therapeutics has highlighted the great importance of precisely designing drug delivery carriers targeting malignant cells, while sparing normal healthy tissues. Despite the enhanced permeation and retention (EPR) effect, the deposition of therapeutic NPs in the tumor microenvironment is often limited by their physicochemical properties such as NP size, molecular weight, surface charge, and critical stromal components including vascular dimensions [1,2,3,4,5]. In addition, nanoparticulate systems must evade the reticuloendothelial system (RES) and renal clearance to achieve preferential accumulation in the tumor microenvironment [4]. RES evasion and prolonged systemic circulation are routinely achieved by grafting polyethylene glycol (PEG) to NPs [6]. Moreover, PEG tagging increases the circulation time of nanocarriers, thereby improving delivery of therapeutics to distant and disseminated tumors [7].

Although EPR-mediated delivery of therapeutics usually facilitates the delivery of NPs in the tumor interstitium, it sometimes does not allow the intracellular delivery of NPs in tumors. This is mainly due to poor interactions with tumor cells and backflow into systemic circulation [8,9]. Therefore, targeting cell surface markers such as α_v_β_3_ integrin, which are crucial for endothelial cell survival during angiogenesis and tumor metastasis, can be an important strategy for targeted drug delivery [10,11]. α_v_β_3_ integrin receptors are generally expressed in many types of cancer cells, such as ovarian and colon cancer cells [12,13]. Cyclic Arg-Gly-Asp peptide (cRGD) was exploited as a targeting ligand, because it selectively binds to α_v_β_3_ integrin [14]. We hypothesized that cloaking RGD peptide in nanocarriers would offer long-term tumor targeting and bioimaging properties, which can further potentiate the theranostic potential of anti-tumor strategies.

Amphiphilic graft copolymers received increased attention in the past decade owing to their potential applications in biosensors and drug delivery [15,16]. Among them, poly(amino acids) are well known to possess excellent biomedical applicability because of their biocompatibility, non-toxic nature, and biodegradability [17]. Poly(succinimide) (PSI) derivatives, such as poly(hydroxyethyl- aspartamide) (PHEA) and poly(aspartic acid), displayed typical amphiphilic characteristics in aqueous environment [18]. Previously, our group successfully investigated the potential applications of PSI derivatives for MRI diagnosis, gene delivery, and image-guided cancer therapy [19,20].

**Figure 1:**
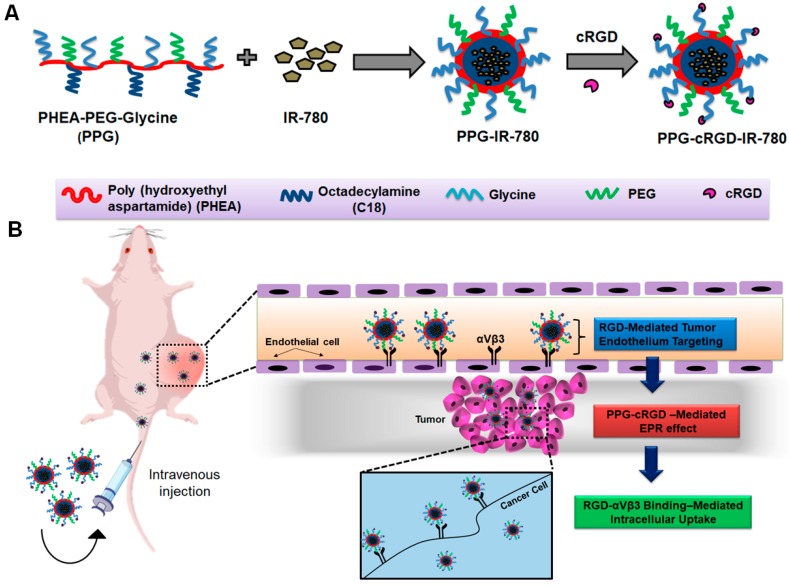
Schematic illustration of long-term tumor targeting and bioimaging mediated by PPG-cRGD NPs. (**A**) Synthesis scheme of PPG-cRGD NPs. (**B**) Tumor homing of intravenously injected PPG-cRGD NPs through RGD-mediated binding to tumor endothelial cells. Intratumorally accumulated PPG-cRGD NPs elicit enhanced intracellular uptake due to specific binding of PPG-cRGD NPs on α_v_β_3_ integrin receptor in tumor cells, resulting in long-term tumor targeting and bioimaging.

In this study, we engineered a precise tumor-targeting nanoconstruct based on amphiphilic PHEA derivative offering the advantages of enhanced systemic circulation, tumor targeting abilities, and intracellular uptake (Figure 1). Polymeric NPs were synthesized from PHEA (P) by grafting hydrophobic octadecylamine (C18), methoxy PEG amine (P), and glycine (G) sequentially onto the PHEA polymeric backbone to produce PHEA-PEG-glycine (PPG) NPs. These PPG NPs were readily formed through self-assembly in aqueous environments and could encapsulate hydrophobic imaging molecules, such as IR-780. PPG NPs were further conjugated with cRGD to obtain tumor-targeting PPG-cRGD NPs via preferential binding to tumor endothelial cells and α_v_β_3_ integrin-expressing cancer cells. The enhanced long-term tumor targeting and imaging abilities of PPG-cRGD NPs were found to be superior to those of non-targeted formulations.

## 2. Results and Discussion

### 2.1. Synthesis of PPG and PPG-cRGD

**Figure 2:**
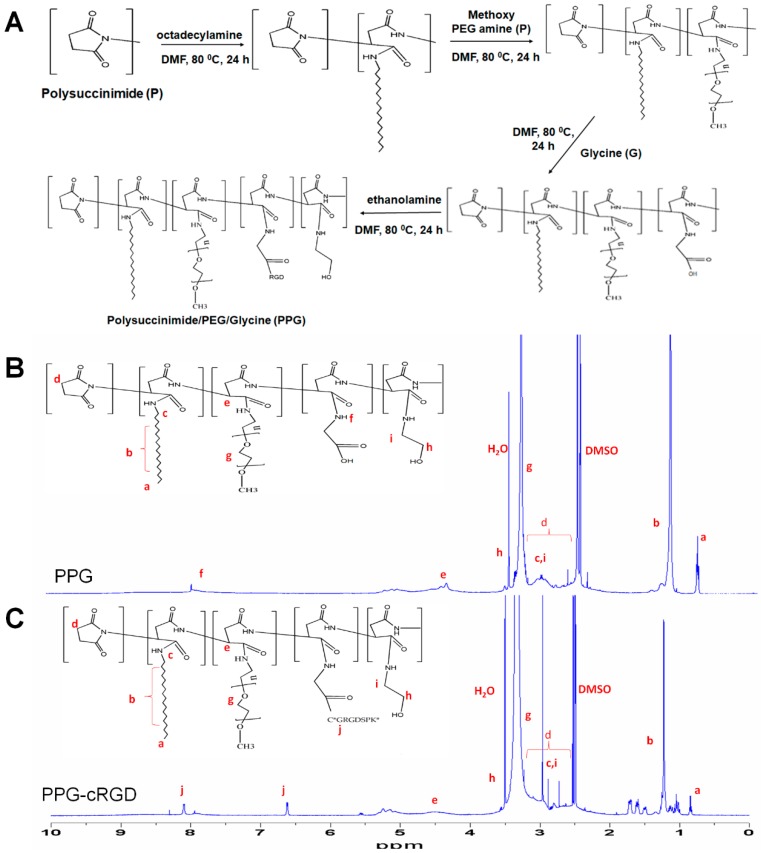
(**A**) Overall reaction scheme of PHEA conjugated with octadecylamine, methoxy PEG amine, and glycine. ^1^H NMR analyses of PPG NPs (**B**) and PPG-cRGD NPs (**C**).

PPG was synthesized using a top-down approach (Figure 2A). First, PHEA-C18 was synthesized by modifying PSI with octadecylamine chains (C18) through a nucleophilic substitution reaction. Then, methoxy PEG amine (10% *w*/*v*) and glycine (15% *w*/*v*) were grafted onto PHEA-C18 through successive aminolysis reactions [18,19] to obtain PPG amphiphilic co-polymer. 

The as-synthesized copolymers readily self-assemble to form micelles in aqueous solution and subsequently PPG NPs. These PPG-NPs were characterized with proton (^1^H) nuclear magnetic resonance (NMR) spectroscopy (Figure 2B). In addition, cRGD was conjugated to PPG NPs to improve the targeting efficiency of the NPs and enhance accumulation in the tumor. cRGD conjugation (PPG-cRGD NPs) was performed by esterification reaction between the carboxyl group of glycine and thiol group of cRGD. The synthesis of PPG-cRGD NPs was also confirmed by ^1^H-NMR as shown in Figure 2C. The signals at 2.5–3.3. ppm corresponded to the methylene proton of PSI [18]. The peak at 0.8 ppm corresponded to methyl protons, those at 1.2–1.5 ppm corresponded to methylene protons, and those at 3 ppm corresponded to methylene protons adjacent to the amino group of C18. The peak at 4.5 ppm could be attributed to methine proton of octadecyl (C18) aspartamide originating from the ring opening reaction of PSI [18]. The signal at 3.5 ppm corresponded to methylene protons of PEG, while the peak at 7.9–8 ppm corresponded to the O=C–NH group of glycine coupled to PHEA [21]. The signals at 6.6–6.7 ppm corresponded to NH_2_ of arginine and those at 7.5–8.3 ppm corresponded to NH of the peptide backbone of cRGD [22]. The degree of substitution of cRGD to PPG was 10 mol %, which was obtained by comparing the characteristic cRGD peaks at 6.6–6.7 ppm with the PPG peak at 4.3–4.7 ppm [23].

### 2.2. Physicochemical Characterization 

**Figure 3:**
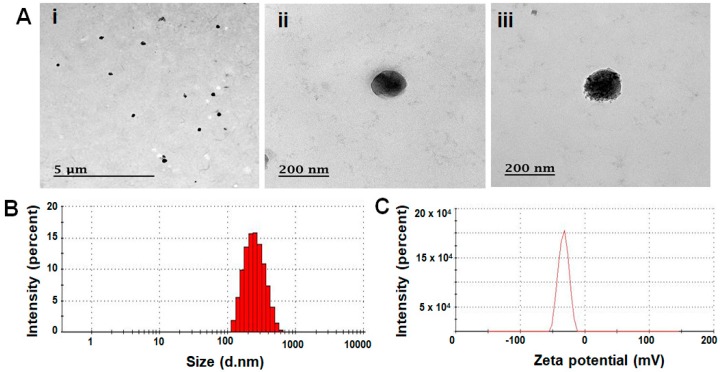
FE-TEM images of PPG-cRGD NPs (**A**i,ii) and PPG-cRGD-IR-780 NPs (**A**iii). (**B**) Hydrodynamic size of PPG-cRGD NPs analyzed by dynamic light scattering. (**C**) Zeta potential of PPG-cRGD NPs measured by Zeta sizer.

The as-synthesized PPG NPs and PPG-cRGD NPs were readily formed by self-assembly in aqueous solution. In this study, we used IR-780, a near infrared (NIR) hydrophobic fluorophore, to visualize the tumor homing of polymeric NPs in real-time imaging. IR-780 can be replaced with hydrophobic therapeutic agents loaded in PPG-cRGD NPs (PPG-cRGD-IR-780) for effective anticancer applications. IR-780 was loaded by a nanoprecipitation technique, with an encapsulation efficiency more than 90% in both the formulations (Table 1). Size distribution of the formulated NPs determines the in vivo fate and their potent biomedical applications. According to field-emission transmission electron microscopy (FE-TEM) images of PPG NPs and PPG-IR-780 NPs, their particle diameters were 168 ± 12 nm and 110 ± 16 nm, respectively (Figure S1). In addition, FE-TEM analyses of PPG-cRGD NPs and PPG-cRGD-IR-780 NPs showed average particle size distribution of 160 ± 21 nm and 146 ± 16 nm, respectively. The as-synthesized NPs were of spherical morphology with good monodispersity (Figure 3A). 

We further evaluated the NP size by dynamic light scattering (DLS) analysis. The NPs followed the same pattern as that observed in FE-TEM analysis, with hydrodynamic sizes ranging between 200–250 nm (Table 1) for all formulations. The hydrodynamic size of the PPG-cRGD-IR-780 found to be 222 ± 3 nm (Figure S2A). Differences in DLS and TEM measurements were attributed to the extension of hydrophilic blocks in DLS analysis. The NPs could encapsulate hydrophobic IR-780 through hydrophobic interactions and condensation, resulting in size reduction after IR-780 loading in PPG or PPG-cRGD NPs. This was evident in FE-TEM and DLS analyses (Table 1). Owing to their size, NPs can preferentially accumulate in the tumor microenvironment due to leaky vasculature and poor lymphatic drainage, which is termed as EPR effect. Besides their inherent EPR effect, PPG NPs were also conjugated with cRGD peptide to enhance the tumor targeting effect. The surface charge of NPs also plays a key role in the systemic circulation of NPs. Despite the optimal size distribution for prolonged systemic circulation, highly positively-charged NPs exhibit nonspecific tissue interactions and undergo phagocytosis leading to lower circulation half-life [24], whereas negatively-charged and neutral NPs exhibit longer circulation and improved accumulation in tumor areas [25]. It was found that the surface charge of all the formulations suspended in aqueous media ranged from −20 to −30 mV (Table 1). The zeta potential of the PPG-cRGD-IR-780 found to be −31 ± 4 mV (Figure S2B). Additionally, we have investigated the potential of PPG-cRGD-IR-780 to improve the long-term circulation and stability by means of their hydrodynamic changes in the plasma. As result depicts (Figure S3), PPG-cRGD-IR-780 evidenced a very minimal size gain after 6 h of incubation showed the reduced non-specific adsorption nature. This property of reduced opsonization further hinders the uptake of NPs by RES system, and thereby enhances the systemic circulation and tumor accumulation. Thus, it was hypothesized that the prepared formulations carrying optimal nanosize range, negative surface charge plasma stability, and tumor-targeting ligand could contribute to enhanced targeting effect.

### 2.3. Critical Micelle Concentration and Stability of PPG NPs 

**Figure 4:**
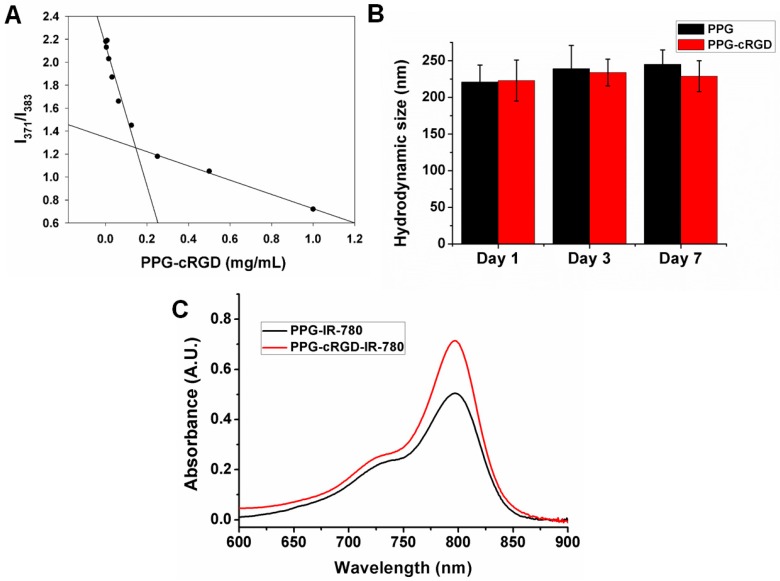
(**A**) Critical micelle concentration of PPG NPs. (**B**) Dynamic light scattering analysis of PPG NPs and PPG-cRGD NPs incubated in phosphate-buffered saline recorded at different time points. (**C**) UV-VIS spectra of PPG-IR-780 NPs and PPG-cRGD-IR-780 NPs depicting characteristic absorbance peak of IR-780.

Amphiphilic polymers self-assemble in aqueous solution to form NPs. NP formation is generally confirmed by determining the minimum concentration of polymer required to form the micellar structure, which is known as the critical micelle concentration (CMC). CMC was evaluated by using a fluorescent dye pyrene and plotting the intensity ratios (I_1_/I_3_) as a function of nanomicelle concentration. The CMC of PPG NPs was found to be 0.143 mg/mL (Figure 4A). Stability of NPs is of prime importance, considering their dynamic nature encountered in in vivo conditions. In this study, the stability of NPs was characterized as a means of their average hydrodynamic diameter over a certain period (Figure 4B). PPG NPs and PPG-cRGD NPs remained stable without any visible aggregation or precipitation in phosphate-buffered saline (PBS) over one week, demonstrating their physiological stability in various media. Furthermore, the physical loading of IR-780 dye into the hydrophobic core of PPG NPs (PPG-IR-780) or PPG-cRGD NPs (PPG-cRGD-IR-780) was evaluated by UV-VIS spectroscopy. As shown in Figure 4C, the characteristic peaks of IR-780 were evident in both the formulations, and the maximum absorbance peak was observed at 798 nm. 

### 2.4. In Vitro Cytocompatibility and Intracellular Uptake 

**Figure 5:**
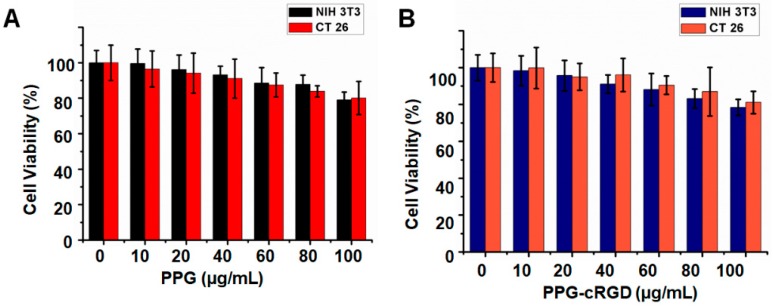
Viability of CT26 cells and NIH 3T3 cells after treatment with PPG NPs (**A**) and PPG-cRGD NPs (**B**) at different concentrations for 24 h. Data are shown as average ± S.D. (*n* = 4).

Biocompatibility of the tumor-targeting formulations is critical for their potential application in anti-tumor therapy. In this study, we evaluated the cytocompatibility of PPG NPs and PPG-cRGD NPs in mouse colon carcinoma cell line (CT26) and normal healthy fibroblast cell line (NIH 3T3). As shown in Figure 5A, the viability of CT26 and NIH 3T3 cells treated with PPG NPs was maintained at around 85–90% even at high concentrations of 100 µg/mL. 

**Figure 6:**
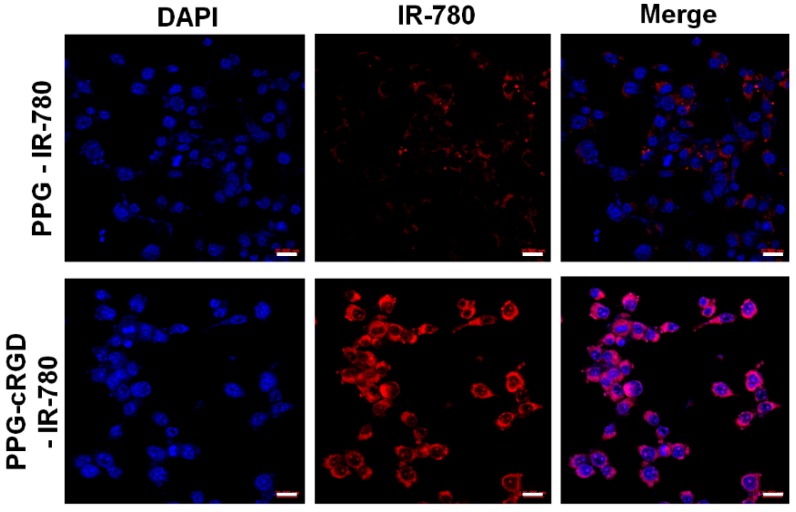
Confocal images of intracellular uptake of PPG-IR-780 NPs and PPG-cRGD-IR-780 NPs after 4-h incubation. The nucleus was stained with DAPI (blue). Scale bar: 20 µm.

Furthermore, cell viability tests showed that both CT26 and NIH 3T3 cell lines treated with PPG-cRGD NPs showed no appreciable cytotoxicity even at high concentrations, indicating the biocompatibility of the synthesized formulations. This result is of importance because the non-toxic nature of NPs can be useful in preventing off-target toxicity. In addition, cellular uptake analysis was conducted in CT26 mouse colon carcinoma cells. As shown in Figure 5, intracellular uptake of PPG-cRGD-IR-780 NPs was significantly increased in CT26 cells, compared with that in PPG-IR-780 NP control. In the targeted group (PPG-cRGD-IR-780 NPs), the bright red fluorescence signal of IR-780 demonstrated enhanced localization of PPG-cRGD-IR-780 NPs in the perinuclear region, whereas weak red fluorescence was observed in the non-targeted PPG-IR-780 NP group (Figure 6). This fluorescence imaging analysis demonstrates the advantage of active targeting of cRGD moiety to cancer cells in which α_v_β_3_ integrin receptors are overexpressed. Additionally, we tested the intracellular uptake of free IR-780 in CT26 cells. Figure S4 indicated that intracellular uptake of free IR-780 was very minimal in the CT26 cells, owing to their hydrophobic nature and poor water solubility. Thus this enhanced intracellular uptake of NPs is one of the crucial parameter in drug delivery applications, where intracellular delivery of therapeutic agents is of utmost concern.

### 2.5. Long-Term Tumor Targeting and Bioimaging 

**Figure 7:**
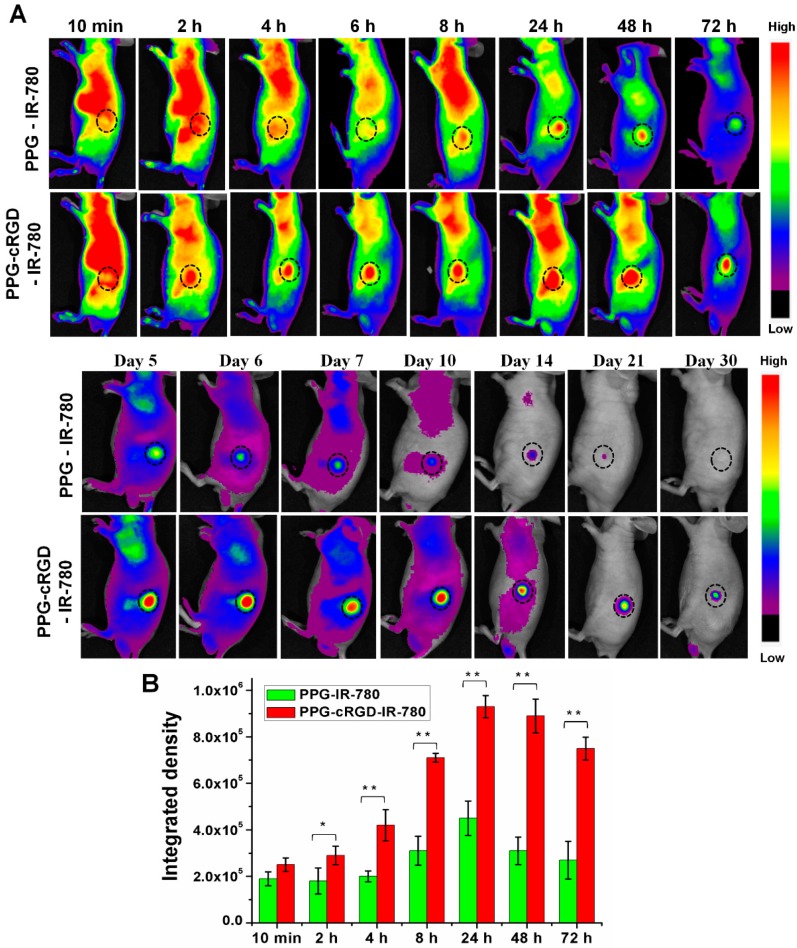
(**A**) Biodistribution analysis of PPG-IR-780 NPs and PPG-cRGD-IR-780 NPs in CT26 tumor-bearing nude mice after intravenous administration. Black circle indicates tumor region. (*n* = 5). (**B**) Fluorescence intensity quantification of tumor region in a time-dependent manner. The data are shown as mean ± SD (*n* = 5). * indicates *p* < 0.05 and ** indicates *p* < 0.01.

Whole body NIR imaging was used to study the biodistribution and tumor targeting of PPG-IR-780 NPs and PPG-cRGD-IR-780 NPs. Figure 7 depicts the fluorescence distribution of PPG-IR-780 NPs and PPG-cRGD-IR-780 NPs injected intravenously into tumor-bearing nude mice. Figure 7A shows that IR-780 fluorescence signals of both PPG NPs and PPG-cRGD NPs were widely distributed throughout the body in the initial 10 min after injection. At 2-h post-injection, PPG-cRGD-IR-780 NPs started to actively accumulate near the tumor owing to the targeting capability of cRGD. At 8-h post-injection, PPG-cRGD NPs exhibited stronger fluorescence signal in the tumor than PPG NPs (Figure 7B), while the accumulated NPs in normal organs were cleared out through the renal route. 

As expected, the tumor accumulation of PPG-cRGD NPs reached its maximum at 24 h post-injection when compared with the PPG NP group and decreased slowly thereafter. Notably, the fluorescence intensity of PPG-cRGD NPs was retained in the tumor region for 21 days, (Figure 7) while that of PPG NPs drastically diminished after day 6. These results indicated that cRGD tagging on PPG NPs aided in enhanced tumor targeting and facilitated longer retention of PPG-cRGD NPs in the tumor region because of the strong binding affinity of cRGD to α_v_β_3_ integrin.

This superior long-term tumor targeting and imaging capabilities can be attributed to the following factors: (1) The size of PPG-cRGD NPs was below 200 nm; at this size, they might not only escape kidney, spleen, and liver filtration, but also accumulate in tumor owing to EPR effect [25]. (2) Negative surface charge of the formulation aided in enhanced residence time and tumor deposition by avoiding non-specific tissue adsorption and retarding the systemic clearance of NPs [2,25]. (3) cRGD offered enhanced tumor endothelium targeting and subsequent cancer cell internalization, resulting in long-term targeting and tracking of tumor region.

**Figure 8:**
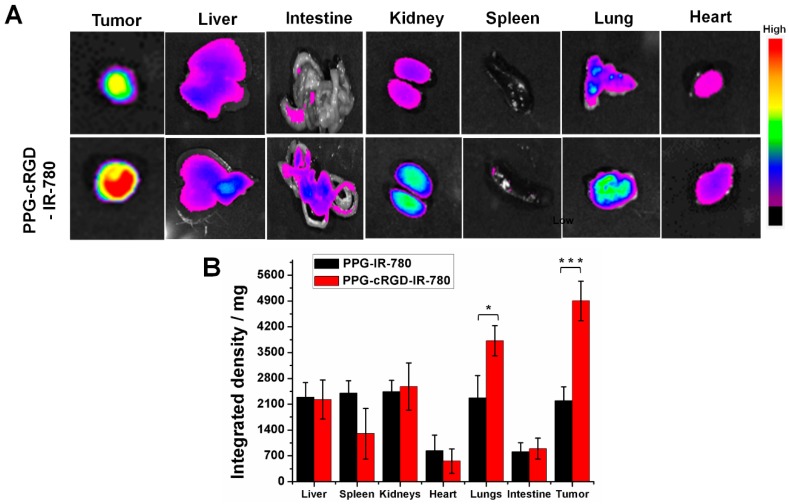
(**A**) Ex vivo fluorescent images of major organs and tumor collected at 72 h post-administration of PPG-IR-780 NPs and PPG-cRGD-IR-780 NPs. (**B**) Quantitative analysis of formulations determined as means of integrated density/mg of organs and tumors. The data are shown as mean ± SD (*n* = 5). * indicates *p* < 0.05 and *** indicates *p* < 0.001.

Ex vivo images of the excised tissues exhibited a stronger fluorescence intensity in the tumor region of PPG-cRGD NPs than that of PPG NPs, indicating excellent tumor targeting ability (Figure 8A,B). Moreover, fluorescence intensity in the lungs and kidneys at 72 h after intravenous injection of PPG-cRGD NPs highly increased, presumably because of the mechanical entrapment of larger-sized NPs in the lung capillaries [26]. However, this may not be a critical problem as the NP composition is based on poly(amino acids), which are known for their superior biocompatibility and non-toxic nature [17]. Furthermore, higher kidney accumulation can be attributed to higher integrin α_v_β_3_ binding affinity in the kidneys [27]. 

**Figure 9:**
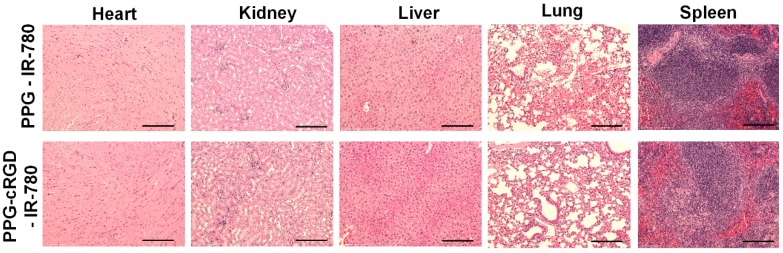
H&E staining of major organs analyzed at 72 h post-injection of nanoparticle formulations. Scale bar: 200 µm.

Histochemical analysis of major organs was performed to evaluate the toxicity of PPG-IR-780 NPs and PPG-cRGD-IR-780 NPs in vivo. The major organs such as the heart, kidney, liver, lung, and spleen were harvested and stained with hematoxylin and eosin (H&E). As shown in Figure 9, no significant tissue damage or inflammation was evident in the organs, which is in accordance with the in vitro cytotoxicity studies. The impressive tumor targeting properties along with excellent biocompatibility reduce toxicity concerns, indicating that PPG could be used as a potential tumor-targeting drug delivery vehicle.

## 3. Materials and Methods 

### 3.1. Materials

PSI was purchased from Desai Chemicals (Shijiazhuang, China). Methoxy-PEG-amine (Mw 750 Da) was purchased from Sun-Bio (Gyeonggi-do, Korea). Diethyl ether, octadecylamine, *N*,*N*-dicyclohexylcarbodiimide (DCC), glycine, and ethanol amine were obtained from Sigma Aldrich (St. Louis, MO, USA). Dimethyl sulfoxide (DMSO) and *N*,*N*-dimethylformamide were obtained from Merck (Darmstadt, Germany). Cyclic RGD (C*GRGDSPK*) was purchased from GenicBio Limited (Shanghai, China). 

### 3.2. Synthesis of PPG and PPG-cRGD NPs

PPG NPs were synthesized according to previously reported protocols with slight modifications [19,20]. Briefly, PSI (3 mmol) was dissolved in dimethylformamide (DMF); C18 (3 mmol) was added in a drop-wise manner and incubated for 24 h at 80 °C in a reflux column. Methoxy PEG amine (10% *w*/*v*) dissolved in DMF was added and incubated for 24 h at 80 °C. Glycine (15% *w*/*v*) dispersed in DMF was added to the product and incubated for 24 h at 80 °C. Finally, excess of ethanolamine (90 µL) was added to the product and reacted for 3 h. Next, the product was precipitated with diethyl ether and dried at room temperature (RT) for 12 h. The samples were dissolved in DMSO and dialyzed (MWCO 3.5 kDa) against water for 3 days. The dialyzed samples were lyophilized and stored in an auto-dry desiccator for future use. PPG-cRGD NPs were synthesized by esterification reaction as reported previously [28,29]. Briefly, PPG (50 mg) was added with 4-dimethylaminopyridine (DMAP) (100 mmol) and cRGD (60 mmol) in 10 mL of DMF. DCC (2 mmol) was added to the reaction mixture and reacted for 5 min at 0 °C and for 4 h at RT. The obtained solution was dialyzed (MWCO 3.5 kDa) against water for 24 h at 4 °C and lyophilized to obtain the final PPG-cRGD powder, which was stored at 4 °C for future use.

### 3.3. Measurement of Particle Size Distribution, Zeta Potential, and Stability

NP size (diameter), polydispersity index (PDI), and surface charge (zeta potential) were determined using a Zetasizer Nano Z instrument (Malvern Instruments, Malvern, UK). Determinations were performed at 25 °C by diluting samples in triple distilled water. PPG NPs or PPG-cRGD NPs (10 mg) were dispersed in PBS (pH 7.4), and the stability of NPs was determined as the means of their size using Zetasizer instrument (*n* = 3).

### 3.4. Nuclear Magnetic Resonance Spectroscopy and Field-Emission Transmission Electron Microscopy 

^1^H NMR spectra of PPG NPs and PPG-cRGD NPs were analyzed by NMR spectrophotometry (300 MHz; Bruker, Billerica, MA, USA). PPG NPs and PPG-cRGD NPs were dissolved in DMSO-*d*_6_. The morphology of PPG NPs and PPG-cRGD NPs was confirmed using TEM (JEM-2000 FX, JEOL, Tokyo, Japan).

### 3.5. Critical Micelle Concentration Determination

The CMC of NPs was determined by a protocol reported previously [30]. Ten µL of pyrene solution was dissolved in acetone (0.45 mg/mL) and allowed to evaporate. Then, 2 mL of aqueous solution of PPG at concentrations ranging from 0.0012 to 1 mg/mL was added to the cuvette containing pyrene. After sonication for 5 min, the mixture was allowed to equilibrate at RT for 24 h. Fluorescence spectra were measured by a microplate reader (Tecan SPARK 10M, Mannedorf, Switzerland) (excitation wavelength: 334 nm). The emission wavelengths at 371 nm (I_1_) and 383 nm (I_3_) were monitored. CMC was calculated by measuring the ratio of I_1_ and I_3_ (I_1_/I_3_). 

### 3.6. IR 780 Loading 

IR-780 loading was carried out by nanoprecipitation method [26]. Twenty mg of NPs were dissolved in 5 mL of distilled water and stirred for 5 min at RT. Then, 0.2 mg of IR-780 dissolved in 1 mL of DMSO was added to PPG solution and sonicated for 5 min. The solution was then dialyzed against water (MWCO 3500) for 12 h and subjected to lyophilization. Drug loading efficiency was calculated by suspending the NPs in DMSO to dissolve the encapsulated drug and to measure the absorbance of the released drug with IR780 standard. Drug loading efficiency of IR 780 was quantified as follows:

Encapsulation Efficiency (EE) = IR 780 weight in NPs/IR 780 weight feed × 100%

Drug loading (DL) = IR 780 weight in NPs/weight of IR 780-loaded NPs × 100%

### 3.7. In Vitro Cytocompatibility and Intracellular Uptake

The mouse colon carcinoma cells (CT26) was obtained from Korean Cell Line Bank. CT26 cells were cultured in DMEM media. Cells were seeded in the 96 well plate with cell density of 4 × 10^4^ cells/well for 12 h. Cells were incubated with pre-determined concentrations of PPG and PPG-cRGD for 24 h. Cytocompatibility was measured by [3-(4,5-dimethylthiazol-2-yl)-5-(3-carboxymethoxy-phenyl)-2-(4-sulfophenyl)-2*H*-tetrazolium] (MTS) assay. MTS reagent was added (20 µL) to the samples and incuabted for 4 h, followed by absorbance measurement at 490 nm. Absorbance was measured by Tecan Spark 10 M multimode microplate reader (Mannedorf, Switzerland) Cytotoxicity was measured by comparing the absorbance values with the non-treated cell controls.

For intracellular uptake, CT26 cells (3 × 10^3^ cells/well) were seeded in a 8-well chamber plate (Lab-Tek, Thermo Fisher Scientific, Waltham, MA, USA) for 12 h in DMEM media. After 12 h, cells were treated for 4 h with the PPG-IR-780 and PPG-cRGD-IR-780 at equivalent concentrations of 5 µg of IR-780/well. Afterwards, cells were washed thrice with PBS and fixed with 4% paraformaldehyde. Cells were then counterstained with the nuclear stain ProLong DAPI (Thermo Fisher Scientific, USA). Fluorescence images were captured with confocal laser scanning microscopy (Zeiss LSM510, Berlin, Germany). 

### 3.8. In Vivo Imaging and Biodistribution Analysis

Animal experiments were conducted according to the regulations of the Ethics Committee of Chonnam National University Medical School and Chonnam National University Hwasun Hospital, South Korea (CNU IACUC-H-2018-59). CT26 tumor was inoculated on the right flank of athymic nude mouse (4 week old, female, *n* = 5). CT26 tumor-bearing nude mice were distributed into PPG-IR-780 NP- and PPG-cRGD-IR-780 NP-treated groups, and administered intravenously with NPs equivalent to 0.5 mg/kg IR-780. Fluorescence imaging was analyzed by optical imaging using a Fluorescence-labelled Organism Bio-imaging Instrument (FOBI, NEO science, Gyeonggi, Korea). At 72 h post-injection, tumors and major organs were dissected and imaged using FOBI system. Fluorescence intensity of the tissues were analyzed as means of integrated density/mg of tissue.

### 3.9. Hematoxylin and Eosin Staining

All major organs, including the lung, spleen, heart, kidneys, and liver, and tumor tissues were fixed with 10% formalin and embedded in paraffin blocks. The tissues (6 µm) were sectioned, dewaxed with xylene, and hydrated with alcohol (ranging from 100%, 95%, 75%, and 50% EtOH) and distilled water. Then, the tissues were stained with H&E.

## 4. Conclusions

This study demonstrated a new strategy to achieve prolonged tumor targeting and enhanced bioimaging capability for poly(aspartamide) NPs. Modulating the physicochemical parameters such as size and surface charge of NPs imparted ideal characteristics to tumor-targeted carriers, including mononuclear phagocyte system evasion and enhanced systemic circulation. In addition, cRGD conjugation to PPG NPs demonstrated enhanced and long-term tumor targeting efficacy and imaging. This enhanced targeting ability is attributed to the specific binding of PPG-cRGD NPS on tumor endothelium and their subsequent internalization into integrin-overexpressing cancer cells. Based on these findings, we propose that the resulting tumor-targeted PPG-cRGD NPs will be useful in potentiating the efficacy of targeted formulations by providing long-term circulation, tumor targeting, and imaging. 

## Figures and Tables

**Table 1:** Physicochemical characteristics of formulations.

Formulation	D/P ^a^ Ratio	Hydrodynamic Size (nm)	Zeta Potential (mV)	Polydispersity Index (PDI) ^b^	EE	DL
PPG	-	235 ± 5	−24 ± 0.8	0.262	-	-
PPG-cRGD	-	238 ± 6	−28 ± 1	0.258	-	-
PPG-IR-780	0.01	219 ± 7	−27 ± 2	0.201	90	1.06
PPG-cRGD-IR-780	0.01	222 ± 3	−31 ± 4	0.232	93	1.23

^a^ D/P ratio = IR 780 weight/NP weight; Data represents mean ± SD, *n* = 3; ^b^ PDI = poly dispersity index.

## Supplementary Materials

The following are available online. Figure S1: FE-TEM images of PPG and PPG-IR-780. Figure S2: DLS analysis of PPG-cRGD-IR-780 NPs. Figure S3: DLS analysis of PPG-cRGD-IR-780 NPs in mouse plasma. Figure S4: Confocal images of free IR-780.
