# Supplementary material for: Design of an Amphiphilic Poly(aspartamide)-Mediated Self-Assembled Nanoconstruct for Long-Term Tumor Targeting and Bioimaging
Source: Molecules. 2019 Mar 2;24(5):885. doi: 10.3390/molecules24050885 (PMC6429357; doi:10.3390/molecules24050885)
Supplement: Supplementary file 1 [file molecules-24-00885-s001.pdf]

Supplementary materials

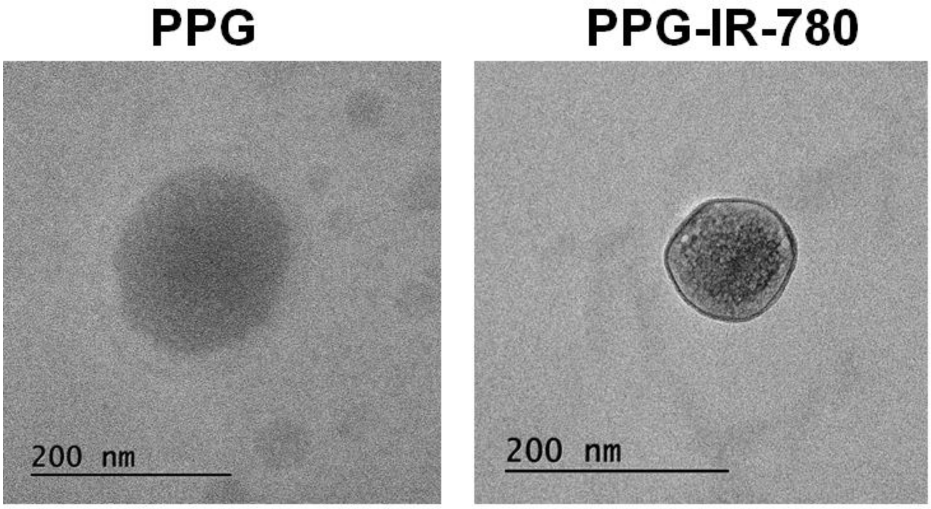

Figure S1. FE-TEM images of PPG and PPG-IR-780 NPs.

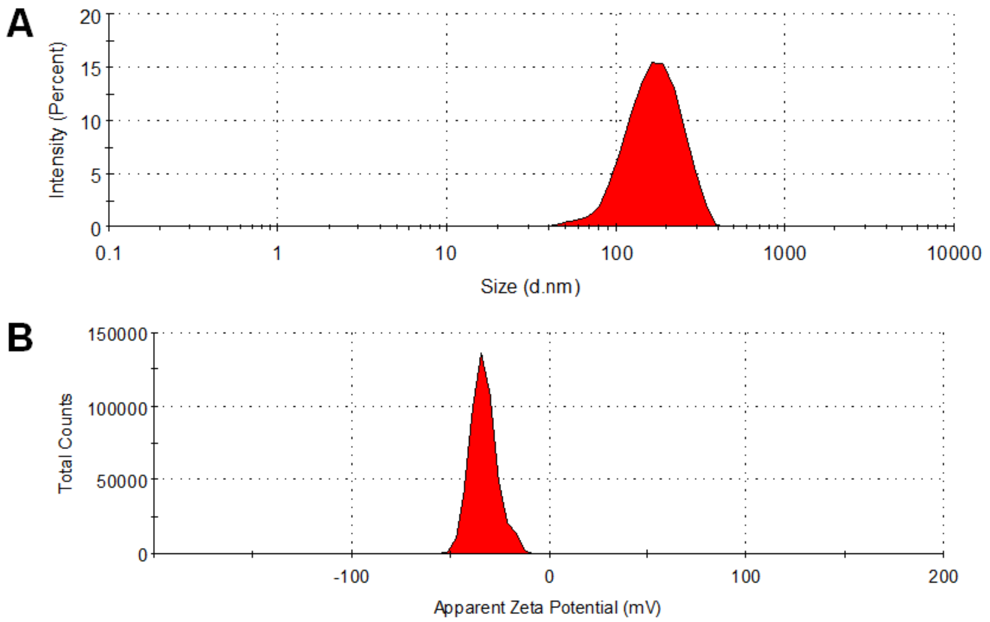

Figure S2. (A) Hydrodynamic size and (B) Zeta potential of PPG-cRGD-IR-780 NPs measured by Zeta sizer.

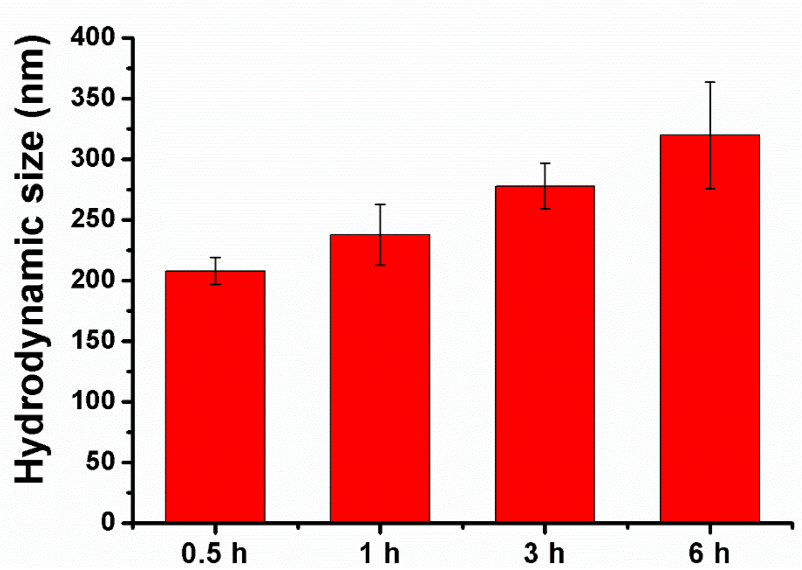

Figure S3. DLS analysis of PPG-cRGD-IR-780 NPs after incubation with 10 % mouse plasma in a time-dependent manner.

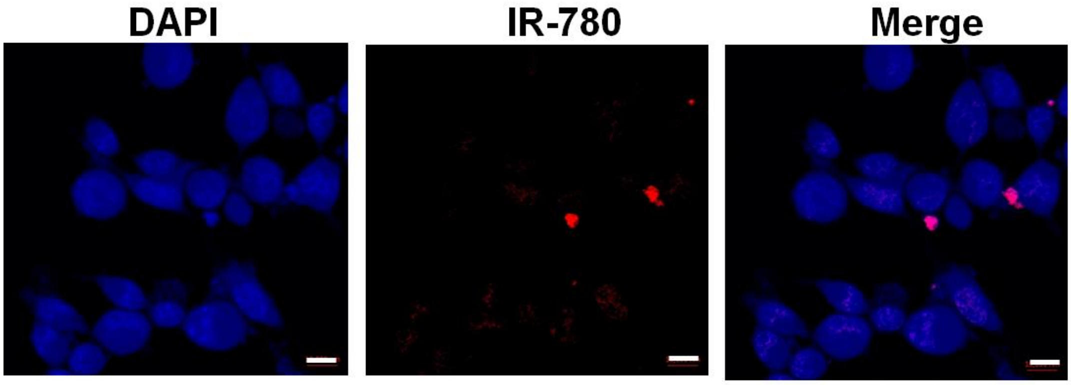

Figure S4. Confocal images of intracellular uptake of IR-780 after 4-h incubation. The nucleus was stained with DAPI (blue). Scale bar: 10 μm.
